# Acute aortic dissection-induced acute respiratory distress syndrome: pathogenesis and clinical implications

**DOI:** 10.3389/fcvm.2025.1654456

**Published:** 2025-11-21

**Authors:** Wen-jun Zhou, Hong-ye Wu, Jia-xuan Wang, Shi-dong Liu, Yun-peng Xu, Bin Song, Jian Liu

**Affiliations:** 1 The First Clinical Medical College of Lanzhou University, Lanzhou, Gansu, China; 2 Department of Cardiovascular Surgery, First Hospital of Lanzhou University, Lanzhou, Gansu, China; 3 Lanzhou University, Lanzhou, Gansu, China; 4 Department of Intensive Care Unit, Gansu Provincial Maternity and Child Health Hospital/Gansu Provincial General Hospital, Lanzhou, Gansu, China

**Keywords:** acute aortic dissection, acute respiratory distress syndrome, lung injury, pathogenesis, diagnosis, intervention

## Abstract

Acute aortic dissection, a life-threatening cardiovascular emergency, is frequently complicated by acute respiratory distress syndrome. This complication exacerbates perioperative risks, poses challenges for clinical management, and impacts patient postoperative recovery. However, a comprehensive understanding of its pathogenesis remains elusive. This review synthesizes evidence to delineate aortic intimal tearing trigger the key initiating events comprising systemic inflammatory response, renin-angiotensin system dysregulation, high mobility group box 1 release, coagulation/fibrinolysis disorder, platelet hyperactivation/consumption, and intestinal ischemia/reperfusion injury. These upstream pathways converge on the lung, inducing injury through sustained inflammation, damage to pulmonary vascular endothelium and alveolar type II epithelial cells, microvascular constriction, microthrombosis, and alveolar fibrin deposition. Notably, crosstalk among some of these pathways may amplify lung injury. By systematically presenting these mechanisms, this review highlights translational opportunities for early diagnosis, monitoring disease progression, and designing targeted therapies to mitigate lung injury and enhance outcomes in these patients.

## 1 Introduction

Acute aortic dissection (AAD) is a critical cardiovascular condition leading to an extremely high mortality rate (1–3). Acute respiratory distress syndrome (ARDS), a common and severe complication in preoperative AAD patients (4–9), poses a significant clinical challenge. According to the latest definition, its diagnosis requires acute hypoxemic respiratory failure (PaO_2_/FiO_2_ ≤ 300 mmHg or SpO_2_/FiO_2_ ≤ 315), bilateral chest imaging opacities, and the exclusion of cardiogenic pulmonary edema (10, 11). Preoperative hypoxemia delays surgery, thereby increasing mortality risk (4, 12), and independently predicts postoperative respiratory dysfunction (13, 14); it also exacerbates the patient's pre-surgical condition, significantly impacting postoperative rehabilitation (15, 16) (Supplementary Table S1). Despite its clinical significance, our current understanding of this condition remains incomplete, primarily due to the lack of large-scale prospective clinical studies and animal models that adequately replicate disease pathophysiology. Current clinical intervention strategies for AAD-induced ARDS lack specificity. This knowledge gap underscores the urgent need to elucidate pathophysiological pathways and identify biomarkers for early diagnosis. This comprehensive literature review aims to reveal the potential mechanisms and clinical intervention strategies of AAD-induced ARDS, thereby providing a reference for early identification, precise prevention and targeted treatment of this disease.

## 2 Material and methods

### 2.1 Literature search and selection

A systematic literature search was conducted from database inception to February 1, 2025, across PubMed, Embase, Web of Science, and the Cochrane Library. The search strategy combined keywords and MeSH terms for “*aortic dissection*, *respiratory distress syndrome*, *acute lung injury*, and *hypoxia*” (full syntax provided in Supplementary Material S1). Study selection followed a PRISMA-guided process (Supplementary Figure S1), with dual independent screening prioritizing clinical relevance and methodological quality.

### 2.2 Definitional framework

The 2024 Global Definition of ARDS (11) is applied to current clinical management and recent studies. Historical studies are referenced using their original terminology [e.g., “acute lung injury (ALI)” from the 1994 American-European Consensus Conference [AECC] criteria], which was later superseded by the Berlin definition.

## 3 Pathogenesis of AAD-induced ARDS

### 3.1 Systemic inflammatory response (SIR)

The SIR represents a critical pathological process in AAD patients (17–20), with the lungs serving as primary target organs through three distinct mechanisms. First, pulmonary capillaries filter circulatory metabolites, bioactive mediators, and particulate matter that accumulate during vascular injury. Second, neutrophil-endothelial interactions induce pulmonary microvascular endothelial cells (PMVECs) apoptosis and extracellular matrix degradation via proteolytic enzyme release, significantly increasing capillary permeability. Third, resident alveolar macrophages amplify local inflammation through pro-inflammatory cytokine cascades when activated by systemic triggers. During SIR, the excessive release of tumor necrosis factor-α (TNF-α), interleukin-6 (IL-6), and interleukin-1β (IL-1β) serves as a key mediator of pathological progression (21). These pro-inflammatory factors activate critical signaling pathways, such as nuclear factor kappa-B (NF-κB) (22), mitogen-activated protein kinase (MAPK) (23), Janus Kinase 2/signal transducer and activator of transcription 3 (24), leading to amplified pulmonary inflammation, injury to pulmonary vascular endothelial cells and alveolar type II epithelial cells (AT2 cells), enhanced vasoconstriction, and microthrombus formation. These synergistic pathological events collectively culminate in the development of ALI.

The SIR is also implicated in the development of AAD-inducedARDS. Experimental evidence from a canine AAD model demonstrates rapid progression of this pathophysiology (25). Within 2 h after the formation of aortic dissection, plasma endotoxin and cytokine levels including TNF-α, IL-6, and interleukin-10 (IL-10) showed significant elevation, progressing to peak concentrations by 6 h. This cytokine surge correlated with progressive hypoxemia (PaO_2_ reduction from 2 to 6 h) and histological confirmation of alveolar edema, hemorrhage, alveolar rupture, alveolar septal broadening, and interstitial broadening. Clinical observations reinforce these experimental findings. AAD patients developing ALI exhibit significantly higher inflammatory markers than those without pulmonary complications [C-reactive protein (CRP) and/or white blood cell count; both *P* < 0.0] (7, 8, 15). Notably, CRP and IL-6 were identified as independent predictors of preoperative hypoxemia, suggesting their utility as early biomarkers (7). Critically, the severity of aortic injury itself, quantified by the percentage of the volume of false lumen to that of the aorta in the descending aorta (AAD%) demonstrated a stronger inverse correlation with PaO_2_/FiO_2_ (*r* = −0.604, *P* < 0.001) and was the sole independent predictor of oxygenation impairment (OR = 1.323, 95% CI = 1.035–1.691, *P* = 0.026). The positive association between AAD% and CRP (*r* = 0.545, *P* < 0.001) further supports aortic structural damage exacerbates systemic inflammation. These findings collectively suggest that ARDS in AAD appears to be closely correlated with the amount of aortic injury, possibly mediated by the magnitude of SIR associated with the aortic injury (6).

### 3.2 Renin-angiotensin system (RAS) dysregulation

The imbalance of RAS homeostasis is involved in the development of ARDS. The activated angiotensin converting enzyme (ACE)-angiotensin (Ang) Ⅱ-Ang Ⅱ type 1 receptor (AT1-R) axis promotes the occurrence of ARDS by inducing an excessive inflammatory response, impairing alveolar barrier function, and triggering dysfunction in the coagulation. While the ACE2-Ang (1–7)-Mas axis induces a negative regulatory effect on the ACE-Ang II-AT1-R axis by degrading Ang II and generating Ang (1–7). Overactivated Ang II disrupts this balance.

Ang II signaling is not only implicated in the pathogenesis of AAD (26), but also plays a pivotal role in driving lung injury associated with this condition (27). Clinical studies demonstrate significantly elevated serum levels of Ang II (28–31), matrix metalloproteinase-9 (MMP-9) (28), and monocyte chemoattractant protein-1 (MCP-1) (29) in AAD patients with ALI compared to controls and non-ALI AAD cases. Postmortem analyses of ALI-complicated AAD patients further reveal upregulated AT1-R, MCP-1 and MMP-9 expression in lung tissues, accompanied by PMVEC apoptosis, infiltration of MMP-9-expressing macrophages, and interstitial edema. Experimental models utilizing β-aminopropionitrile (BAPN)-induced aortic pathology with Ang II infusion recapitulate these features, showing aggravated lung injury with macrophage accumulation and MMP-9 overexpression compared to BAPN-only controls.

**Figure 1:**
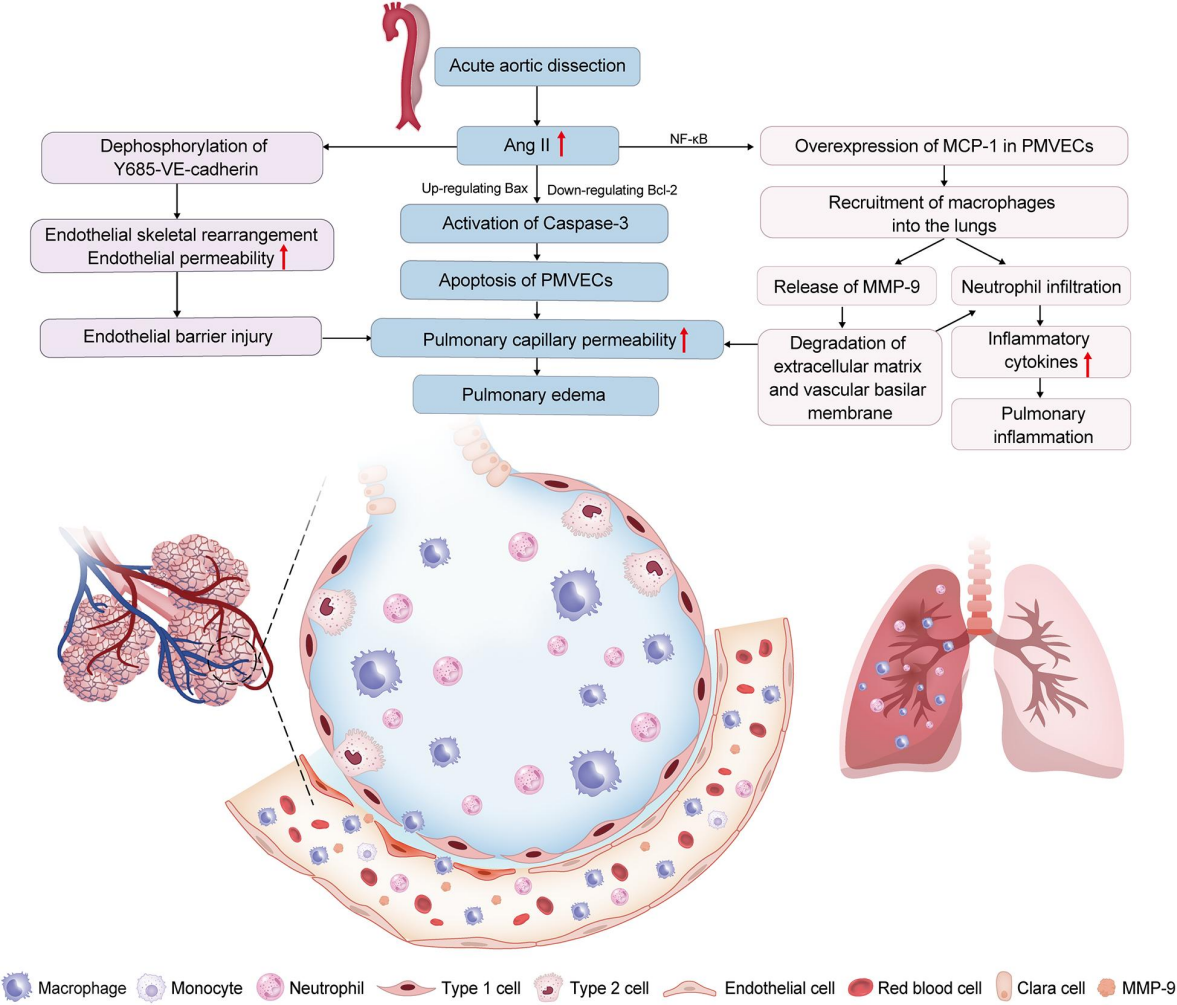
Renin-angiotensin system (RAS) dysregulation is involved in acute aortic dissection (AAD)-induced acute lung injury (ALI). Increased angiotensin II (Ang II) in AAD patients leads to the excessive expression of monocyte chemoattractant protein-1 (MCP-1) in pulmonary microvascular endothelial cells (PMVECs); through nuclear factor-kappa B (NF-κB) pathway, inducing the recruitment of macrophages into the lungs. Increased. Macrophages in the lungs have high matrix metalloproteinase 9 (MMP-9) expression, which increases the pulmonary capillary permeability, and aggravates neutrophil infiltration. Ang II induces apoptosis of PMVECs by the down-regulation of the B-cell lymphoma 2 (Bcl-2) protein and the up-regulation of the BCL-associated X (Bax) protein, further activating caspase-3. Ang II induces the apoptosis and cytoskeletal rearrangement of PMVECs by dephosphorylation of Y685-vascular endothelial cadherin (Y685-VE-cadherin). These mechanisms result in ALI by disrupting the pulmonary vascular endothelial barrier and amplifying pulmonary inflammation.

Mechanistic studies reveal Ang II drives lung injury through two parallel pathways: AT1-R-mediated macrophage recruitment (demonstrated by attenuated pulmonary infiltration following AT1-R blockade despite persistent MMP-9 elevation) and NF-κB-dependent upregulation of MCP-1 in PMVECs, which drives chemotaxis of MMP-9-expressing macrophages (28, 29). Macrophages recruited via these pathways exacerbate vascular permeability through MMP-9-mediated endothelial damage and neutrophil infiltration (32). Concurrently, upregulated MCP-1 induces PMVECs apoptosis via B-cell lymphoma-2 (Bcl-2)/BCL-associated X (Bax) imbalance and caspase-3 activation, further compromising pulmonary microvascular integrity (29). Although the aforementioned studies did not delineate the relative contributions of Ang II-AT1-R binding and MCP-1 elevation in driving MMP-9-expressing macrophages recruitment to the lungs, establishing MCP-1 and MMP-9 as key mediators of Ang II-driven lung injury during the preoperative phase of AAD supports their potential as early predictive biomarkers and therapeutic targets. In addition, Wu et al. found that the expression level of vascular endothelial cadherin (VE-cadherin) is closely related to the apoptosis and skeletal rearrangement of PMVECs induced by Ang II, while the dephosphorylation of Y685-VE-cadherin is involved in Ang Ⅱ-mediated pulmonary microvascular endothelial barrier injury (30, 33). These discoveries also sheds new light on the mechanisms and potential interventions for AAD-inducedARDS (Figure 1).

### 3.3 High mobility group box 1 protein (HMGB1) release

HMGB1 is a nuclear nonhistone protein with a potent pro-inflammatory effect, secreted from the nucleus to the extracellular environment through the non-classical or passive release under stress response (34). It binds to the receptor for advanced glycation end products (RAGE) and activates the MAPK signalling pathway. The phosphorylation of the downstream p38MAPK and the activation of NF-κB lead to the release of several pro-inflammatory factors (35). The activation of the HMGB1/RAGE signalling pathway is a pivotal link in the inflammatory cascade and a crucial mediator of lung injury induced by various factors (36, 37).

Elevated serum HMGB1 levels are associated with both AAD progression and the development of AAD-inducedARDS. At 24 h post-admission, plasma levels of HMGB1 and RAGE were significantly elevated in all AAD patients compared to controls (*P* < 0.05), with notably higher concentrations in the ALI subgroup than in the non-ALI (*P* < 0.05). During the 96-hour monitoring period, both groups exhibited a progressive increase in HMGB1/RAGE levels alongside a concurrent decline in PaO_2_/FiO_2_ ratios. The ALI patients consistently demonstrated more severe dysregulation in these parameters at all timepoints (*P* < 0.05). Spearman analysis revealed strong negative correlations between the PaO_2_/FiO_2_ ratio and both HMGB1 (*r* = −0.978) and RAGE (*r* = −0.944), while HMGB1 and RAGE themselves showed a strong positive correlation (*P* < 0.001 for all) (38). These findings suggest that dynamic monitoring of HMGB1/RAGE trajectories provides critical prognostic insights for ALI risk stratification in AAD. However, this study did not comprehensively investigate the mechanisms underlying the elevated HMGB1 levels observed in the ALI subgroup compared to non-ALI subjects. Beyond the fundamental pathological processes of AAD, additional pivotal factors may contribute to HMGB-drivenARDS. This hypothesis is supported by evidence showing HMGB1's involvement in alternative lung injury pathways triggered by distinct etiological mechanisms. For instance, intestinal I/R injury, a potential secondary complication of AAD, has been shown to induce necroptotic enterocyte-derived HMGB1 release, promoting lung injury through neutrophilic inflammation and neutrophil extracellular trap formation via the Toll-like receptor 4/myeloid differentiation factor 88 (TLR4/MyD88) signaling pathway (39). The coexistence of such multifactorial mechanisms underscores the complexity of HMGB-driven ARDS pathogenesis in AAD patients, necessitating a more holistic approach to fully elucidate these mechanisms.

### 3.4 Coagulation/fibrinolysis disorder

**Figure 2:**
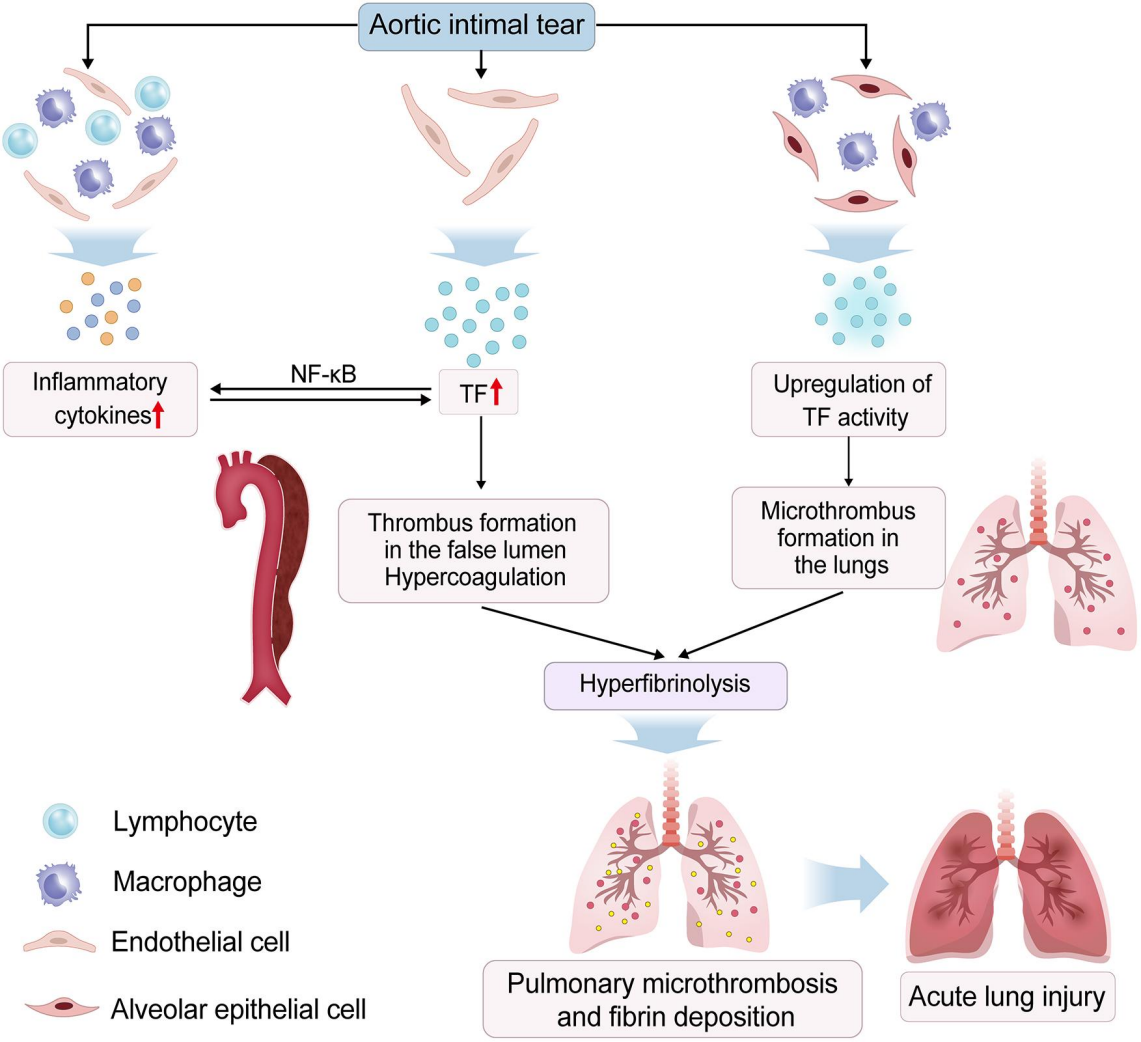
Massive tissue factors (TFs) release is involved in acute aortic dissection (AAD)-induced acute lung injury (ALI). Aortic intimal tear results in the massive release of tissue factors (TFs) by endothelial cells. TF induces production of inflammatory factors by endothelial cells. Increased inflammatory factors also increases the secretion of TFs. TFs initiates the extrinsic coagulation pathway, inducing thrombus formation in the false lumens, subsequently triggering hyperfibrinolysis, which may result in pulmonary microthrombosis and fibrin deposition. Furthermore, alveolar epithelial cells and macrophages activate the coagulation process in the alveoli by upregulating TF activity. These mechanisms contribute to ALI through enhancing pulmonary microthrombosis and alveolar fibrin deposition, as well as amplifying pulmonary inflammation.

During aortic intimal tear in AAD, blood directly contacts the aortic media's extracellular matrix, triggering a defense response. This results in the release of inflammatory cytokines by immune cells and endothelial cells, along with tissue factor (TF) from endothelial cells due to vascular injury. Excessive TF not only induces a great production of inflammatory factors by endothelial cells, but also initiates the extrinsic coagulation pathway, the latter inducing extensive thrombus formation in the false lumens, subsequently triggering hyperfibrinolysis (40). These processes may lead to pulmonary microthrombosis and alveolar fibrin deposition (41, 42). The excessive release of inflammatory factors further increases the secretion of TF by activating the NF-κB pathway, leading to the occurrence of the coagulation cascade (41, 43). Alveolar epithelial cells and alveolar macrophages (AMs) also activate the coagulation process in the alveoli by upregulating TF activity (44, 45). These intricate and intersecting mechanisms associated with TF are involved in the pathogenesis of AAD-induced lung injury (Figure 2). Gao et al. reported that significantly increased levels of TF (*F* = 133.67, *P* < 0.001; *F* = 68.14, *P* < 0.001) and tissue factors pathway inhibitor (TFPI) (*F* = 31.98, *P* < 0.001; *F* = 45.58, *P* < 0.001) in both serum and bronchoalveolar lavage fluid (BALF) among preoperative Stanford type-A aortic dissection (ST-AAD) patients with ALI compared to those without. Furthermore, a significant association exists between oxygenation impairment and levels of TF in both serum and BALF in preoperative AAD patients (40). Despite the elevation in TFPI concentration, it remains much lower than that of TF (about 1% of TF's concentration). This may deplete the TFPI pool in endothelial cells, leaving insufficient TFPI to neutralize TF and reverse its coagulation activation.

Plasminogen activator inhibitor-1 (PAI-1), the predominant fibrinolysis inhibitor through plasmin suppression, drives lung injury through various mechanisms including the following. PAI-1 promotes fibrin deposition and αIIbβ3 integrin-dependent platelet aggregation, forming prothrombotic niches (46, 47). Additionally, PAI-1 enhances pulmonary inflammation by regulating inflammatory signaling pathways such as MyD88/NF-κB activation and interleukin-1 receptor-associated kinase M/suppressor of cytokine signaling 1 suppression (48, 49). Moreover, elevated PAI-1 level is also associated with endothelial barrier dysfunction and the development of pulmonary edema (50). Preoperative ST-AAD patients with ALI have persistently higher levels of PAI-1 in serum and BALF compared to those without ALI (*F* = 213.88, *P* < 0.001; *F* = 107.95, *P* < 0.001). The concentration of PAI-1 in BALF is significantly higher than that in the serum. Moreover, a significant association is present between PAI-1 levels in BALF and oxygenation impairment among patients with ST-AAD (40). The aforementioned results uncovered a pertinent phenomenon: in ST-AAD patients, systemic hyperfibrinolysis coexists with the inhibition of fibrinolysis due to elevated levels of PAI-1. However, the impact of pulmonary PAI-1 on oxygenation is primarily mediated through its localized upregulation, as evidenced by two key findings: (1) Lung-derived PAI-1 from alveolar cells (including macrophages and epithelial cells) directly promotes fibrin deposition in pulmonary tissue (47, 51); (2) Targeted anticoagulation through inhaled heparin effectively reduces pulmonary fibrin formation and microvascular thrombosis (52, 53). This spatial specificity confirms the predominant role of local pulmonary mechanisms rather than systemic effects.

Notably, this prospective study is primarily limited by its small sample size, single-center design, and the lack of BALF data at specific timepoints, which may have omitted valuable insights. Consequently, while the findings of this study provided evidence suggesting an association between elevated TF and PAI-1 levels and AAD-induced ARDS, their prognostic value remains to be validated in larger, multi-center cohorts.

### 3.5 Platelet hyperactivation/consumption

Qin et al. (54) found that AAD canine models possess increased mean platelet volume (MPV)/platelet count and platelet size distribution width, indicating the presence of activated platelet. They performed the dynamic monitoring of both platelet activation levels and systemic inflammatory markers, identifying the simultaneous onset of platelet activation and systemic inflammation. Furthermore, activated platelets are involved in the occurrence of post-dissection inflammation (55). Patients with AAD also show a decreased platelet count (56, 57). Patients with a shorter duration of disease onset show a lower platelet count upon admission, especially within 3 days. Following aortic intimal injury, platelets are the first responders adhering to disrupted endothelial surfaces, with subsequent leukocyte recruitment contributing to false lumen thrombosis (58, 59). This process leads to subsequent consumption of platelets. Activated platelets mediate thrombo-inflammation through dual modulation of coagulation cascades and inflammatory signaling, establishing a self-perpetuating pathological milieu characteristic of aortic dissection progression.

Clinical studies suggest platelet hyperactivation post-AAD may contribute to hypoxemia and ALI. A single-center, prospective cohort study revealed that, compared with the low MPV/platelet count group, patients in the high MPV/platelet count group had a significantly higher risk of hypoxemia (all *P* < 0.05) (59). Pan et al. (60) revealed an increase in platelet-mediated thromboxane B2 (TXB2) production and corresponding changes in the prostaglandin I2 (PGI2)/TXB2 ratio in patients with AAD who develop ALI. Moreover, the imbalance in the above ratio is an independent risk factor associated with preoperative ALI in AAD. Platelets undergo activation after the initiation of AAD and subsequently release thromboxane A2 (TXA2), while TXB2 serves as the stable metabolite of TXA2. The production of TXA2 induces platelet aggregation and vasoconstriction, whereas PGI2 works in the opposite manner. Therefore, the excessively activated platelets after AAD onset may enhance coagulation by releasing TXA2, thereby contributing to lung injury. Notably, activated platelets are involved in the procoagulant process through various mechanisms, with TXA2 being just one of them. Whether additional procoagulant mechanisms mediated by platelet activation contribute to the development of AAD-induced ARDS requires further investigation.

In addition, activated platelets exacerbate lung inflammation by interacting with endothelial cells, and immune cells [such as polymorphonuclear leukocytes (PMNs) and monocytes] via pro-inflammatory mediators (61, 62), thereby amplifying alveolar-capillary barrier damage and vascular permeability (63). When platelet counts decrease, the loss of platelet-derived protective signals impairs endothelial barrier integrity, leading to fluid and protein leakage in both pulmonary and systemic microvasculature (64). Although platelet-mediated mechanisms of pulmonary inflammatory injury have been well documented in lung injury induced by multiple etiologies (65, 66), there is currently not enough evidence (preclinical or clinical) demonstrating their direct involvement in AAD-induced ARDS. In addition, due to methodological constraints (e.g., studies combining surgically/non-surgically managed patients with undocumented hypoxemia timelines) (59), current clinical evidence linking activated platelets to AAD-induced ARDS requires further identification.

### 3.6 Intestinal ischemia-reperfusion (I/R) injury

Organ hypoperfusion occurs when an aortic dissection involves branch arteries. The rigorous blood pressure control measures are aimed at preventing further deterioration of aortic dissection, potentially exacerbating tissue and/or organ malperfusion in patients with chronic hypertension who have adapted to the state of hyper-perfusion. The systemic hypoperfusion associated with AAD, particularly when the dissection involves critical vascular branches including the celiac artery, superior mesenteric artery, and inferior mesenteric artery, contributes to the development of intestinal I/R injury (67).

**Figure 3:**
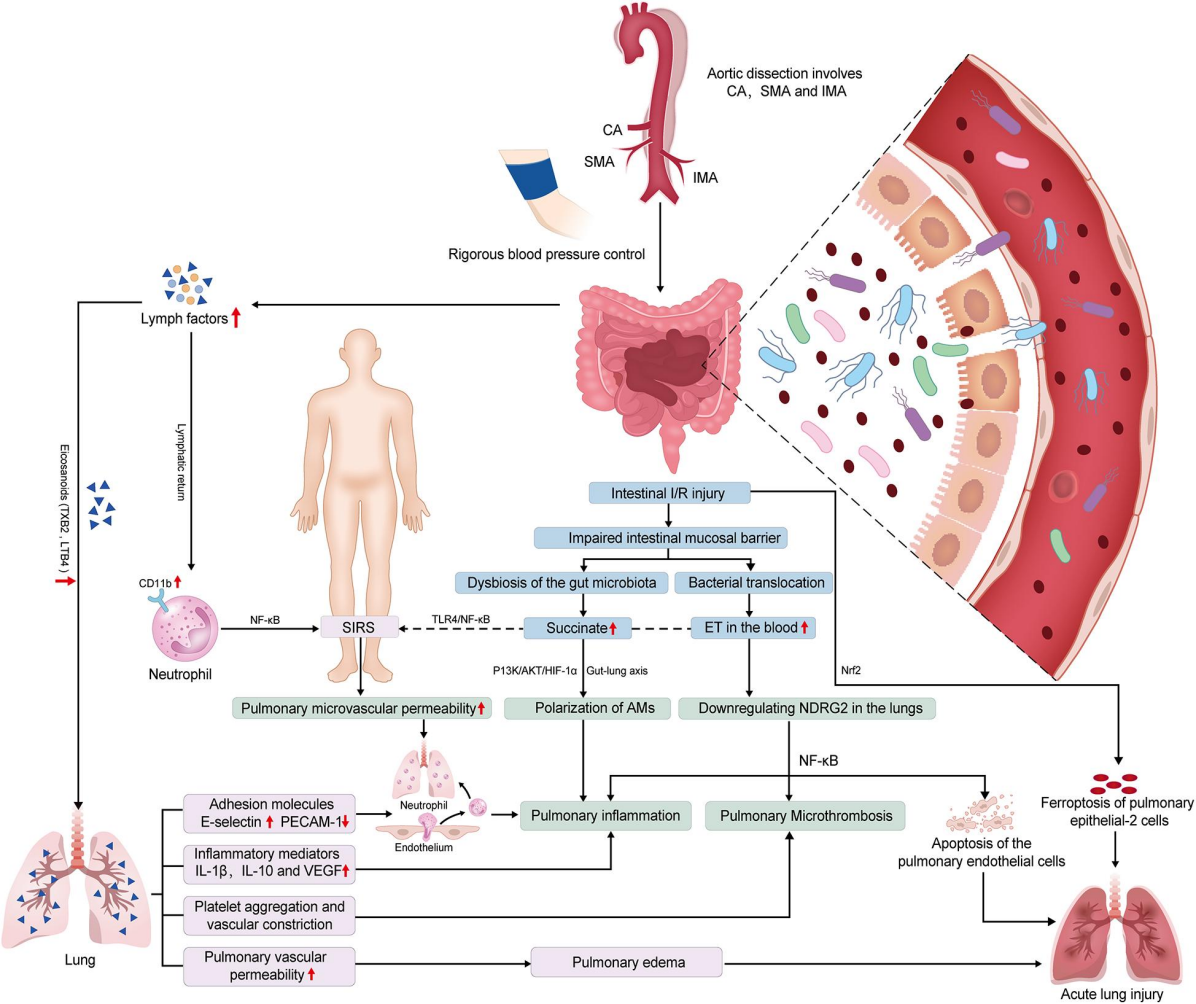
Potential mechanisms of intestinal ischemia/reperfusion (I/R)-induced acute lung injury (ALI) in preoperative acute aortic dissection (AAD) patients. The systemic hypoperfusion associated with AAD, particularly when the dissection involves celiac artery (CA), superior mesenteric artery (SMA), and inferior mesenteric artery (IMA), contributes to intestinal I/R injury. Subsequently, the occurrence of ALI may be induced through synergistic interactions among the following pathways. (1) Gut-lung inflammatory axis: Intestinal I/R triggers endotoxin-mediated Toll-like receptor 4 (TLR4)/nuclear factor kappa-B (NF-κB) activation, driving systemic inflammation (SIR) and pulmonary endothelial apoptosis via N-Myc downstream-regulated gene 2 (NDRG2) downregulation, with concomitant coagulation cascade amplification. (2) Microbiota-metabolite interplay: Dysbiosis-induced succinate accumulation promotes alveolar macrophage polarization through succinate receptor 1 (SUCNR1)-dependent phosphatidylinositol 3-kinase (PI3K)/AKT kinase (AKT)/hypoxia-inducible factor 1-α (HIF-1α) signaling, exacerbating lung injury. (3) Lymphatic mediation: Mesenteric lymph transports key mediators to the lung, including eicosanoids leukotriene B4 (LTB4) and thromboxane B2 (TXB2), as well as cytokines interleukin-1β (IL-1β) and vascular endothelial growth factor (VEGF). These mediators upregulate adhesion molecules E-selectin and platelet endothelial cell adhesion molecule-1 (PECAM-1), promoting vascular hyperpermeability and platelet activation. Circulating lymph upregulates neutrophil cluster of differentiation 11b (CD11b) and pro-inflammatory mediators, inducing pulmonary microvascular hyperpermeability and neutrophilic infiltration. (4) Epithelial ferroptosis: Nuclear factor erythroid 2-related factor 2 (Nrf2)-regulated ferroptosis in alveolar epithelial type II cells (AT2 cell) contributes to pulmonary barrier dysfunction.

Intestinal I/R initiates systemic and lung injury through interconnected pathways. First, disruption of the intestinal mucosal barrier facilitates bacterial/endotoxin translocation into systemic circulation, activating the TLR4/NF-κB pathway to amplify systemic inflammation (68, 69). Concurrently, intestinal I/R downregulates N-Myc downstream-regulated gene 2 in pulmonary endothelial cells, exacerbating apoptosis and NF-κB-driven inflammatory cascades (70). The gut-lung axis further contributes via microbiota dysbiosis and succinate imbalance. Elevated levels of succinate accumulates in the lungs, promoting M1-polarization of AMs through succinate receptor 1-mediated activation of the phosphoinositide 3-kinase (PI3K)/protein kinase B (Akt)/hypoxia-inducible factor 1-alpha (HIF-1α) pathway, thereby aggravating lung injury (71). Second, mesenteric lymph transports pro-inflammatory mediators like eicosanoids (leukotriene B4, TXB2) and cytokines, upregulating endothelial E-selectin and boosting leukocyte adhesion. TXB2 also triggers platelet aggregation and vasoconstriction, worsening pulmonary microvascular permeability and edema (72, 73). These mediators also activate systemic NF-κB signaling, upregulating neutrophil CD11b expression and cytokine production, ultimately driving neutrophil infiltration and microvascular dysfunction (74). Emerging evidence suggests ferroptosis of AT2 cells, mediated by NF-E2-related factor 2 signalling, contributes to intestinal I/R-induced lung injury *in vivo* (75). Collectively, the aforementioned mechanisms ultimately contribute to the development of systemic inflammatory response syndrome and lung injury through the synergistic effects of persistent inflammation, microthrombosis, vascular leakage, and cell death (Figure 3).

While the mechanisms of intestinal I/R injury contributing to lung injuryhave been elucidated in animal models and *in vitro* studies, these findings have yet to be further validated in experimental AAD models or more clinical mechanistic investigations. Previous studies have suggested an association between aortic dissection involving the celiac trunk/mesenteric arteries and preoperative hypoxemia (7), potentially mediated by gut-originated inflammation. Furthermore, emerging researches have demonstrated that intestinal barrier dysfunction occurring subsequent to AAD is implicated in SIR and lung injury providing preliminary insights into the interplay mechanism between gut and lung injuries following AAD (68, 76, 77). However, the scientific validity and methodological rigor of the aforementioned study design and its conclusions warrant critical scrutiny. Notable limitations include the absence of controlled exclusion of confounding factors (e.g., systemic hypoperfusion, direct gastrointestinal vascular compromise by dissection) and incomplete assessment of I/R injury biomarkers (e.g., oxidative stress markers, lactate, creatine kinase, lactate dehydrogenase). Consequently, the temporal relationships and pathophysiological interplay among SIR, intestinal injury, and lung injury following AAD require further mechanistic exploration.

## 4 Uniqueness and heterogeneity in pathogenesis

**Figure 4:**
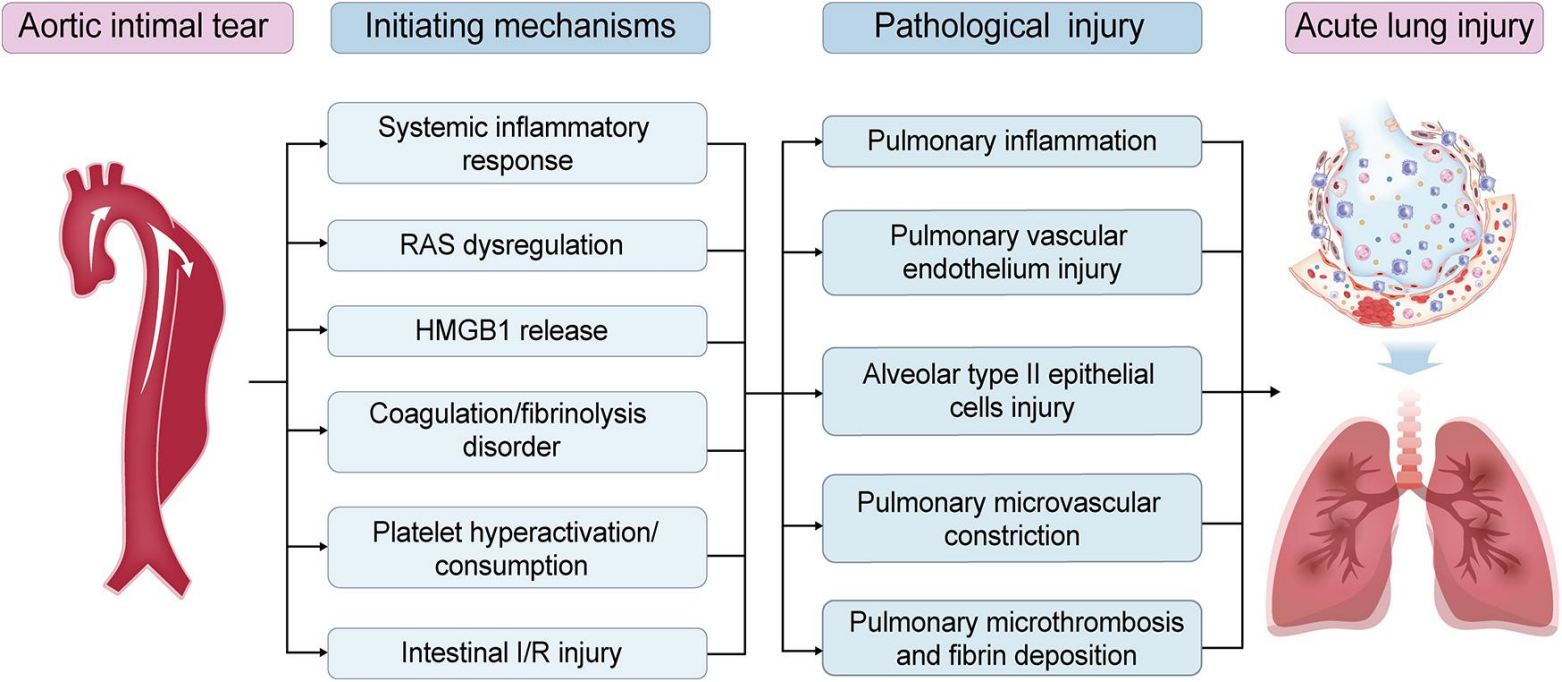
Uniqueness in pathogenesis of acute aortic dissection (AAD)-induced acute lung injury (ALI). The pathogenesis of AAD-induced ALI represents a highly complex process involving intricate interactions across multiple hierarchical levels and diverse mechanistic pathways. It is characterized by a distinct precipitating factor, the aortic intimal tear, followed by diverse initiating mechanisms. These mechanisms encompass systemic inflammatory response (SIR), renin-angiotensin system (RAS) dysregulation, high mobility group box 1 (HMGB1) release, coagulation/fibrinolysis dysregulation, platelet hyperactivation/consumption, and intestinal ischemia/reperfusion (I/R) injury. Within these pathways, key mediators contribute to amplifying pulmonary inflammation, damaging vascular endothelium, impairing alveolar type II epithelial cells (AT2 cells), promoting microvascular constriction, enhancing microthrombosis and alveolar fibrin deposition, ultimately culminating in ALI.

Patients with AAD exhibit a high incidence of preoperative ALI (34.9%–51.0%) (8, 78), a condition now encompassed by the modern ARDS definition. In experimental models, AAD-induced lung injury frequently manifests immediately post-dissection onset with rapid progression (25). The pathogenesis of AAD-induced lung injury involves intricate interactions across multiple mechanistic pathways. This multi-mechanism synergy gives rise to a distinct form of ARDS. Different from ARDS associated with other etiologies, AAD-induced ARDS is characterized by a distinct precipitating factor, the aortic intimal tear, followed by diverse initiating mechanisms. These mechanisms encompass SIR, RAS activation, HMGB1 release, coagulation/fibrinolysis dysregulation, platelet hyperactivation/consumption, and intestinal I/R injury. Within these pathways, key mediators (e.g., MCP-1, MMP-9, TF) contribute to amplifying pulmonary inflammation, damaging vascular endothelium, impairing AT2 cells, promoting microvascular constriction, enhancing microthrombosis and alveolar fibrin deposition, ultimately culminating in AAD-inducedlung injury (Figure 4). Potential cross-talk between mechanisms, such as SIR exacerbating coagulation abnormalities and HMGB1 amplifying SIR, creates a self-perpetuating lung injury cycle. Notably, the contribution of these mechanisms vary depending on the anatomical involvement (e.g., ascending vs. descending aorta), extent of dissection, true lumen perfusion status, and underlying comorbidities (6).

Studies have reported significantly higher rates of preoperative ALI are observed in ST-AAD (41.5%–53.8%) (40, 60) compared to Stanford type-B aortic dissection (ST-BAD) (23.7%) (16). This clinical disparity likely originates from distinct pathophysiological features. ST-AAD involves the ascending aorta, with potential extension either retrogradely towards the proximal segments (aortic root), or anterogradely towards the distal segments (aortic arch, and even descending aorta). While, ST-BAD dissection originates distal to the left subclavian artery in the descending aorta and extends further downwards (1). Anatomical location disparities (ascending aorta vs. descending aorta) not only determine the scope of hemodynamic alterations but also drive differential lung injury mechanisms. Ascending aortic dissection can directly compress the left main bronchus causing regional hypoxia (79), while simultaneously elevating the expression of M2-like macrophages by involving the pericardial tissue, thereby exacerbating SIR (80). In contrast, descending aortic dissection predominantly involves the celiac artery and mesenteric vasculature, preferentially aggravating lung injury through gut-derived endotoxin translocation. Furthermore, the extent of dissection and true lumen perfusion status collectively create an “ischemia-reperfusion gradient”. In summary, the pathological network of AAD-induced ARDS exhibits significant spatial heterogeneity and temporal dynamics, requiring the establishment of a multidimensional hierarchical research framework in future investigations.

## 5 Clinical translation and application

The urgency of aortic dissection repair and the limited preoperative optimization window present challenges for perioperative management of AAD-inducedARDS. It is well-established that unpredictable aortic rupture remains the most critical threat to AAD patients regardless of ARDS status, and surgical intervention persists as the primary therapeutic approach (81). However, immediate implementation is not universally feasible due to specific complications (e.g., abnormally elevated troponin) and constraints on healthcare resources. Notably, AAD-induced ARDS demonstrates substantial prevalence, exacerbates disease severity, and significantly compromises postoperative recovery and long-term prognosis. Within the narrow preoperative and intraoperative windows, prioritized multidisciplinary evaluation and targeted interventions are essential to achieve early diagnosis, precise risk stratification, and effective clinical management of AAD-induced ARDS. To the best of our knowledge, no established clinical management guidelines currently exist for AAD-induced ARDS.

### 5.1 Early diagnosis and monitoring

Currently, there are no specific biomarkers recommended for the early identification and diagnosis of AAD-induced ARDS. Based on the latest guidelines for ARDS, a diagnosis of AAD-induced ARDS should be considered when the following conditions are met: (1) Precipitated by AAD onset; (2) Acute onset or worsening of hypoxemic respiratory failure within 1 week of AAD onset; (3) Using the PaO_2_/FiO_2_ ≤ 300 mmHg or pulse oximetry SpO_2_/FiO_2_ ≤ 315 to identify hypoxemia; (4) Pulmonary edema is not primarily attributable to either cardiogenic factors (e.g., from left ventricular dysfunction or aortic regurgitation) or fluid overload; and hypoxemia/gas exchange abnormalities are not primarily attributable to atelectasis; (5) Bilateral infiltrates on chest radiography and computed tomography or bilateral B lines and/or consolidations on ultrasound. (6) Exclusion criteria were ARDS induced by other causes, and exacerbation of chronic lung diseases (10, 11).

Apart from diagnostic criteria, various monitoring methods are essential for clinical assessment, including respiratory status (respiratory rate and depth), pulse oximetry and/or arterial blood gas analysis (PaO_2_/FiO_2_ or SpO_2_/FiO_2_ ratios), computed tomography angiography and non-contrast chest CT (demonstrating the “dual signs” of aortic dissection and ground-glass opacities), and cardiopulmonary ultrasound (assessing cardiac structure/function and pulmonary B-lines). A review of the existing literature suggests that several readily available and cost-effective biomarkers show promise for early identification. These include markers of inflammation (e.g., AAD%, CRP and IL-6) (6, 7), and platelet activation (MPV/platelet count ratio) (59). Although preliminary evidence from single-center, small-scale studies suggests that some biomarkers (e.g., Ang II, MCP-1, MMP-9, HMGB1/RAGE, TF, PAI-1, TXB2 and PGI2/TXB2 ratio) may have value in identifying patients and stratifying risk, their translation to routine clinical screening remains limited by the challenges of standardization, cost, and the need for validation in larger, prospective cohorts (28–30, 38, 40, 60). Beyond these, biomarkers like AT1-R and VE-cadherin (including pY685-VE-cadherin), while mechanistically linked to AAD-induced lung injury in preclinical models, represent a more nascent area of research (30, 33) (Supplementary Table S2). From a different methodological perspective, recent advances in serum metabolomics, derived from studies of ALI, provide novel insights into investigating the pathogenesis and potential predictors for AAD-induced lung injury (82).

### 5.2 Intervention strategies

#### 5.2.1 Respiration assistance

For AAD patients with ARDS, non-invasive ventilation is the most widely utilized clinical approach, primarily aimed at alleviating patients’ dyspnea and improving oxygenation indices. Clinicians can select the most suitable form of non-invasive ventilation based on the patient's condition, including nasal cannula, mask oxygen therapy, Venturi mask, high-flow nasal cannula therapy, and non-invasive positive pressure ventilation (NIPPV) (83). A recent study excluding patients with cardiogenic edema demonstrated that preoperative NIPPV significantly improved outcomes in AAD patients with preoperative hypoxemia, markedly reducing ED mortality from 25.67% to 8.33% (*P* = 0.002). It confirmed improved 3-day and 5-day survival (*P* = 0.03 and *P* = 0.001, respectively) and identified NIPPV as an independent protective factor against 3-day (adjusted HR 0.102, *P* = 0.03) and 5-day mortality (adjusted HR 0.057, *P* = 0.005). Furthermore, compared with conventional oxygen therapy, the NIPPV group had a significantly lower ED intubation rate (2.78%, *P* = 0.004) and significantly shorter durations of postoperative ventilation, ICU stay, and hospital stay (all *P* < 0.001) (83). It should be noted that this study has a single-center design, which may introduce selection bias and warrants future validation by multi-center prospective studies. While prone positioning has not been studied in AAD with ARDS, and its potential risks (e.g., to hemodynamics and cannulation sites) warrant strict case-by-case consideration. When non-invasive ventilation fails, invasive mechanical ventilation with a lung-protective strategy (low tidal volume, limited plateau pressure, titrated PEEP) is essential, despite potential hemodynamic impacts. For the most critically ill patients, extracorporeal membrane oxygenation can be considered as a salvage option. However, the requisite systemic anticoagulation confers a high bleeding risk in AAD patients, mandating rigorous multidisciplinary evaluation to balance potential survival benefits against life-threatening complications. Currently, high-quality evidence for these advanced strategies remains limited.

#### 5.2.2 Medical treatment

Based on previous research evidence, therapeutic targeting of both pathways reducing false lumen burden and suppressing inflammation may be an effective intervention target for AAD-induced ARDS (6). At the most established end of the evidence spectrum are pharmacological interventions with support from human clinical trials. For instance, preoperative ulinastatin (300,000 IU) administration is associated with reduced postoperative hypoxemia in patients with ST-AAD (7); while sivelestat, which is approved for SIRS-associated ARDS in some regions (84, 85), has been shown to improve oxygenation and reduce inflammatory biomarkers in these patients (86), and its potential benefits are being further assessed in an ongoing clinical trial (NCT05874700). Furthermore, the synergistic attenuation of SIRS by ulinastatin and sivelestat through complementary mechanisms suggests a new therapeutic paradigm for pharmacological intervention (87). As a cornerstone of the medical management for AAD, beta-blockers reduce heart rate and blood pressure, thereby lowering aortic wall shear stress. Notably, Yusuke Jo et al. demonstrated that early beta-blocker administration (within 24 h post-AAD onset) mitigates systemic inflammation and improves the PaO_2_/FiO_2_ ratio (88). Furthermore, restoring impaired intestinal barrier function may also help mitigate AAD-associated SIR and lung injury (68, 76). However, large-scale randomized controlled studies specifically addressing preoperative ARDS in AAD are still needed to establish definitive therapeutic guidelines for these therapeutic methods.

Beyond established clinical interventions, preclinical studies have revealed several potential pharmacological agents for mitigating AAD-induced lung injury. AT1-R blocking agent (Valsartan) reduces macrophage accumulation and alleviates lung injury, whereas downstream MMP inhibition mitigates injury without altering macrophage influx, which underscores the therapeutic superiority of targeting the upstream AT1-R (28). Bindarit, an MCP-1 inhibitor, alleviates Ang II-induced ALI by suppressing NF-κB-mediated MCP-1 production in hPMVECs, thereby limiting macrophage recruitment (89). In addition, it directly protects these cells from apoptosis by regulating the Bax/Bcl-2 ratio and caspase-3 activation (29). In mouse models confirmed that IL-22 alleviates Ang II-induced ALI, and further cellular assays revealed that this protective effect is mediated through inhibition of the JAK2/STAT3 signaling pathway, thereby reducing apoptosis in PMVECs (78). At the regulatory RNA level, MiR-145-5p reduces Ang II-induced ACE2 shedding and the inflammatory response in alveolar epithelial cells by targeting a disintegrin and metalloprotease 17 (ADAM17) and inhibiting the AT1R/ADAM17 pathway (90). Beyond these pathways, inhibition of the HMGB1 pathway, an emerging strategy with demonstrated efficacy in preclinical models of sepsis and intestinal I/R-related ALI (39, 91), represents another potential avenue for disrupting upstream inflammatory cascades in AAD. It is crucial to emphasize that the evidence for all these targets is derived from animal models and *in vitro* systems, which cannot fully replicate human pathophysiology. This translational gap necessitates future studies that prioritize validation in human lung tissues or more physiologically relevant animal models. Investigating combinatorial approaches (e.g., co-targeting MCP-1 and ADAM17) and evaluating the translational potential of advanced modalities like miR-145-5p or IL-22-based biologics are critical next steps (Table 1).

**Table 1:** Candidate therapies in acute aortic dissection-induced acute lung injury/acute respiratory distress syndrome.

Target	Representative drugs	Mechanism of action	Research status	Ref/Trial No.
Serine protease	Ulinastatin	Inhibits multiple serine proteases	Clinical	(7)
Neutrophil elastase	Sivelestat	Inhibits neutrophil elastase	Clinical	(86); NCT05874700
Beta-1 adrenergic receptor	Beta-blocker	Mitigates systemic inflammation via reduction of aortic mechanical stress and blockade of catecholamine effects.	Clinical	(88)
AT1-R	ARBs (Valsartan)	Inhibits AT1-R mediated Ang II-induced macrophage recruitment to the lung.	Preclinical	(28)
MCP-1	Bindarit	Inhibits MCP-1-mediated PMVECs apoptosis. Inhibits Ang II-induced MCP-1 overexpression via modulating NF-κB pathway; Inhibits MCP-1-mediated macrophage infiltration.	Preclinical	(29, 89)
JAK2/STAT3	IL-22	Inhibits PMVECs apoptosis.	Preclinical	(78)
AT1-R/ADAM17	MiR-145-5p	Reduces Ang II-induced ACE2 shedding and the inflammatory response.	Preclinical	(90)

MCP-1, monocyte chemoattractant protein-1; PMVECs, pulmonary microvascular endothelial cells; AT1-R, angiotensin II type 1 receptor; ARBs, angiotensin II receptor blockers; JAK2/STAT3, Janus kinase 2/signal transducer and activator of transcription 3; ADAM17, a disintegrin and metalloprotease 17; Ang II, angiotensin II; ACE2, angiotensin-converting enzyme 2.

## 6 Conclusions

ARDS is a prevalent preoperative complication that significantly challenges perioperative management and impedes patient recovery. Despite growing research interest, robust evidence remains scarce. This scarcity stems primarily from limitations in current clinical studies, which are often single-center (a design that may introduce selection bias and limit generalizability), retrospective, and have small sample sizes along with the historical application of inconsistent diagnostic criteria (e.g., ALI,). Basic research also faces challenges, frequently relying on simplified models. SIR and RAS activation have emerged as the most extensively studied mechanisms with high clinical translation potential. Although studies suggest that platelet hyperactivation-driven inflammatory lung injury and intestinal I/R-driven lung injury may be involved, the direct causal relationship between these mechanisms and AAD-induced lung injury still requires further validation. Critical knowledge gaps persist regarding mechanistic interactions, including SIR, gut barrier dysfunction and lung injury crosstalk, gut-lung axis signaling, as well as endothelial/immune/epithelial cell interplay. Future research priorities should focus on delineating the dynamic evolution, core mechanisms, regulatory targets, and cross-species conserved pathways of AAD-induced ARDS through integrated multicenter prospective cohort studies and pathophysiologically relevant animal models. Additionally, pathways with clinical translation potential, such as SIR, coagulation-fibrinolysis imbalance, and gut-lung axis signaling, require rigorous validation via multi-omics integrative analyses. Key approaches should include serial serum proteomic and metabolomic profiling, prospective thromboelastography monitoring, and gut microbiome-metabolome mapping. Collectively, these efforts will contribute to elucidating pivotal regulatory mechanisms and will establish a theoretical foundation for early detection and targeted intervention of this complication.
