# Supplementary material for: Acute aortic dissection-induced acute respiratory distress syndrome: pathogenesis and clinical implications
Source: Front Cardiovasc Med. 2025 Nov 21;12:1654456. doi: 10.3389/fcvm.2025.1654456 (PMC12679279; doi:10.3389/fcvm.2025.1654456)
Supplement: Supplementary file 1 [file Table1.docx]

**Supplemental TABLE 1. Clinical studies on preoperative acute respiratory distress syndrome in acute aortic dissection (n ≥50)**

| **Study** | **Design** | **Monocentric or multi‑ centric** | **Research objects** | **Patients numbers** | **Morbidity** | **Impacting postoperative rehabilitation** | **Ref** |  |  |  |
| --- | --- | --- | --- | --- | --- | --- | --- | --- | --- | --- |
| Duan, X. Z, et al. 2018 | Retrospective | Monocentric | ST-AAD | 172 | 44.80% | Not reported | (7) |  |  |  |
| Sugano Y, et al.2005 | | | | Prospective | Monocentric | AAD | 61 | 51.00% | Not reported | (8) |
| Xia, L, et al. 2024 | Retrospective | Monocentric | ST-AAD | 229 | 49.00% | Not reported | (9) |  |  |  |
| Teng Cai, et al. 2023 | Retrospective | Monocentric | AAD | 212 | 35.80% | Not reported | (13) |  |  |  |
| Guo, Z, et al. 2019 | | | Retrospective | Monocentric | ST-AAD | 505 | 46.50% | Hypoxemia group with longer median of intubation time, longer ICU stay, longer hospital stay, lower activity of daily living scale score (both P<0.01) | (15] |  |
| Tamura Y, et al. 2021 | Retrospective | Monocentric | ST-BAD | 224 | 23.70% | Hypoxemia group with longer ICU and hospital stays (median 20 vs. 16 days, p < 0.05 and median 7 vs. 5 days, p < 0.01) | (16) |  |  |  |
| Zeng, Z, et al. 2021 | | Prospective | Monocentric | AAD | 96 | 39.58% | Not reported | (38) |  |  |
| Gao, Z, et al. 2019 | | Prospective | Monocentric | ST-AAD | 53 | 41.50% | Hypoxemia group with longer mechanical ventilation time (27.24±8.37 vs. 17.33±7.36 h, P<0.01), longer ICU stay (42.27±10.85 vs. 33.45±9.05 h, P<0.01), and longer hospital stay (17.77±5.00 vs. 13.48±3.97 days, P<0.01). | (40) |  |  |
| Pan, X, et al. 2018 | Prospective | Monocentric | ST-AAD | 130 | 53.80% | Not reported | (60) |  |  |  |
| Ren, W, et al. 2019 | Retrospective | Monocentric | AAD | 621 | 34.90% | Not reported | (78) |  |  |  |

AAD, acute aortic dissection, ST-AAD, Stanford type-A aortic dissection; ST-BAD, Stanford type-B aortic dissection
