# Supplementary material for: Acute aortic dissection-induced acute respiratory distress syndrome: pathogenesis and clinical implications
Source: Front Cardiovasc Med. 2025 Nov 21;12:1654456. doi: 10.3389/fcvm.2025.1654456 (PMC12679279; doi:10.3389/fcvm.2025.1654456)
Supplement: Supplementary file 2 [file Table2.docx]

**Supplemental TABLE 2 Candidate biomarkers for acute aortic dissection-induced acute lung injury/acute respiratory distress syndrome (Part 1 of 5)**

| **Biomarker** | **Pathway / Mechanism** | **Specimen** | **Direction** | **Key Reported Findings** | **Diagnostic / Prognostic performance** | **Study Design/ Level of evidence** | **Ref.** |
| --- | --- | --- | --- | --- | --- | --- | --- |
| AAD% (Imaging biomarker) | Associated with AAD-Hypox risk; putative mechanisms include aortic injury extent (AAD%) driving systemic inflammatory response, leading to hypoxemia. | **Clin:** CTA + 3DReconstruction | ↑ | Significantly higher AAD% in AAD-Hypox vs. AAD-nonHypox group (*p* < 0.001). Significant negative correlation with PaO₂/FiO₂ (r = -0.604; *p* <0.001). AAD% was an independent predictor of PaO₂/FiO₂ (OR=1.323, 95%CI=1.035-1.691, *p* =0.026) | **Prognostic.** Independently predicted AAD-Hypox. | **Clinical:** Level 2b (OCEBM) | (6) |
| CRP | Associated with AAD-Hypox risk; putative mechanisms include systemic inflammatory response. | **Clin:** Patient serum | **↑** | CRP was an independent predictor of AAD-Hypox (OR=1.034, 95% CI=1.008–1.061, *p* =0.010). | **Prognostic.** Independently predicted AAD-Hypox. | **Clinical:** Level 3b (OCEBM) | (7) |
| IL-6 | Associated with AAD-Hypox risk; putative mechanisms include systemic inflammatory response. | **Clin:** Patient serum | ↑ | IL-6 was an independent predictor of AAD-Hypox (OR=1.050, 95% CI=1.003–1.100, *p* =0.036). | **Prognostic.** Independently predicted AAD-Hypox. | **Clinical:** Level 3b (OCEBM) | (7) |
| Ang II | Ang II induces oxidative stress and PMVECs apoptosis, resulting in increased pulmonary microvascular permeability, edema, and inflammation. | **Clin:** Patient serum | ↑ | Elevated Ang II in AAD-ALI vs. AAD-nonALI (*p* < 0.05). | **Prognostic.** Predicts increased AAD-ALI risk. | **Clinical:** Level 2a (OCEBM) | (28-30) |
|  |  | **Pre:** Rat lung tissue; | ↑ | BAPN+Ang II group: marked pulmonary inflammation and alveolar exudates. Increased MDA & W/D ratio; decreased SOD (BAPN+Ang II vs. BAPN/control, *p* < 0.01). | Not applicable | **Preclinical** (Animal model) | (28) |

AAD%, the percentage of the volume of false lumen to that of the aorta in the descending aorta; AAD, acute aortic dissection; AAD-Hypox, acute aortic dissection patients with hypoxemia; AAD-nonHypox, acute aortic dissection patients without hypoxemia; CTA, computed tomographic angiography; OCEBM, Oxford Centre for Evidence-Based Medicine; CRP, C-reactive protein; AAD-ALI, acute aortic dissection with acute lung injury; IL-6, Interleukin-6; Ang II, angiotensin II; PMVECs, pulmonary microvascular endothelial cells; AAD-ALI acute aortic dissection patients with acute lung injury; AAD-nonALI, acute aortic dissection patients without acute lung injury; BAPN, Beta-aminopropionitrile; MDA, malondialdehyde; W/D ratio, wet/dry weight ratio; SOD, superoxide dismutase.

**Supplemental TABLE 2 Candidate biomarkers for acute aortic dissection-induced acute lung injury/acute respiratory distress syndrome (Part 2 of 5)**

| **Biomarker** | | **Pathway / Mechanism** | **Specimen** | **Direction** | **Key Reported Findings** | **Diagnostic / Prognostic performance** | **Study Design/ Level of evidence** | **Ref,** |
| --- | --- | --- | --- | --- | --- | --- | --- | --- |
| Ang II | Ang II induces oxidative stress and PMVECs apoptosis, resulting in increased pulmonary microvascular permeability, edema, and inflammation. | **Pre:** Mouse lung tissue | ↑ | Elevated pulmonary microvascular permeability and lung W/D ratio (Ang II vs control, *p* < 0.01; | Not applicable | **Preclinical** (Animal model) | (30), |  |
|  |  | **Pre:** Mouse lung tissue | ↑ | Significant pulmonary edema, and abundant inflammatory cell infiltration (p < 0.05 vs control). | Not applicable | **Preclinical** (Animal model) | (78) |  |
|  |  | **Pre:** Rat PMVECs/ mice PMVECs | ↑ | Enhanced PMVECs apoptosis (Ang II group, TEM findings). | Not applicable | **Preclinical** (In vitro) | (29-30), (78) |  |
| MMP-9 | Ang II/ Macrophage-derived: MMP-9 Macrophage-derived MMP-9 mediates Ang II-induced lung injury by promoting oxidative stress, pulmonary inflammation and edema. | **Clin:** Patient serum | ↑ | Elevated MMP-9 in AAD-ALI vs. AAD-nonALI (*p* < 0.05). | **Prognostic.** Predicts increased AAD-ALI risk. | **Clinical:** Level 2a (OCEBM) | (28） |  |
|  |  | **Pre:** Human post-mortem lung tissue | ↑ | Immunofluorescence identified CD68+ macrophages as the major MMP-9 source in AAD-ALI. | Not applicable | **Preclinical** (Translational, observational) | (28） |  |
|  |  | **Pre:** Rat lung tissue | ↑ | MMP-9 upregulation (mRNA/protein) concurrent with pulmonary edema and inflammatory cell infiltration in BAPN+Ang II group. MMP-9 antagonist attenuated lung injury and oxidative stress (↓MDA, ↑SOD, ↓W/D ratio). | Not applicable | **Preclinical** (Animal model) | (28） |  |

Ang II, angiotensin II; PMVECs, pulmonary microvascular endothelial cells; W/D ratio, wet/dry weight ratio; TEM, transmission electron microscopy; MMP-9, matrix metalloproteinase-9; OCEBM, Oxford Centre for Evidence-Based Medicine; AAD-ALI, acute aortic dissection with acute lung injury; MDA malondialdehyde; SOD, superoxide dismutase; W/D ratio, wet/dry weight ratio

**Supplemental TABLE 2 Candidate biomarkers for acute aortic dissection-induced acute lung injury/acute respiratory distress syndrome (Part 3 of 5)**

| **Biomarker** | **Pathway / Mechanism** | **Specimen** | **Direction** | **Key Reported Findings** | **Diagnostic / Prognostic performance** | **Study Design/ Level of evidence** | **Ref.** |
| --- | --- | --- | --- | --- | --- | --- | --- |
| MCP-1 | Ang II/ MCP-1 MCP-1 mediates Ang II-induced hPMVECs apoptosis via upregulation of Caspase-3/Bax and downregulation of Bcl-2. | **Clin:** Patient serum | ↑ | Elevated MCP-1 in AAD-ALI vs. AAD-nonALI (*p* < 0.05). | **Prognostic.** Predicts increased AAD-ALI risk. | **Clinical:** Level 2a (OCEBM) | (29） |
|  |  | **Pre:** Human post-mortem lung tissue | ↑ | Enhanced MCP-1 immunostaining (AAD-ALI vs. control)  Increased hPMVECs apoptosis (AAD-ALI vs. control) | Not applicable | **Preclinical** (Translational, observational) | (29） |
|  |  | **Pre:** hPMVECs (A549 cells) | ↑ | Increased MCP-1 mRNA & protein (Ang II vs control, *p* < 0.01). MCP-1-dependent regulation of Bcl-2 (↓*p* <0.01 vs. control), Bax (↑*p* <0.01), and Caspase-3 (↑*p* <0.01); reversed by Bindarit (*p* <0.05 vs. Ang II). | Not applicable | **Preclinical** (In vitro) | (29） |
| AT1-R | Ang II/ AT1-R AT1-R mediates Ang II-induced macrophage recruitment to the lung. | **Pre:** Postmortem lung tissue | ↑ | Enhanced AT1-R immunostaining (AAD-ALI vs. control) | Not applicable | **Preclinical** (Translational, observational) | (29） |
|  |  | **Pre:** Rat lung tissue |  | Pharmacological evidence shows macrophage recruitment is an AT1-R-dependent, MMP-9-independent process.  AT1-R blocking reduces macrophage accumulation. | Not applicable | **Preclinical** (Animal model) | (30） |

MCP-1, monocyte chemoattractant protein-1; Ang II, angiotensin II; hPMVEC, human pulmonary microvascular endothelial cells; Bax, BCL-associated X Protein; Bcl-2, B-cell lymphoma-2; AAD-ALI, acute aortic dissection with acute lung injury; AAD-nonALI, acute aortic dissection patients without acute lung injury; OCEBM, Oxford Centre for Evidence-Based Medicine; AT1-R, angiotensin II type 1 receptor; MMP-9, matrix metalloproteinase-9.

**Supplemental TABLE 2 Candidate biomarkers for acute aortic dissection-induced acute lung injury/acute respiratory distress syndrome (Part 4 of 5)**

| **Biomarker** | **Pathway / Mechanism** | **Specimen** | **Direction** | **Key Reported Findings** | **Diagnostic / Prognostic performance** | **Study Design/ Level of evidence** | **Ref.** |
| --- | --- | --- | --- | --- | --- | --- | --- |
| **VE-cadherin** | Ang II/ VE-cadherin VE-cadherin downregulation mediates Ang II-induced endothelial barrier dysfunction via downregulation, enhanced apoptosis, and cytoskeletal rearrangement. | **Pre:** Mouse lung tissues; Rat PMVECs | ↓ | Decreased VE-cadherin expression in vivo (*P* < 0.05 vs. control/sham) and in vitro (*P* < 0.05 vs. control). | Not applicable | **Preclinical** (Animal & In vitro) | (33） |
| pY685-VE-cadherin | Ang II/ pY685-VE-cadherin Y685-VE-cadherin dephosphorylation mediates Ang II-induced pulmonary endothelial barrier injury. | **Pre:** Mouse lung tissues; Rat PMVECs | ↓ | Decreased pY685-VE-cadherin (Ang II vs control, *p* < 0.05), contributing to cytoskeletal rearrangement and elevated permeability. | Not applicable | **Preclinical** (Animal & In vitro) | (30) |
|  |  |  |  |  |  |  |  |
| HMGB1 & RAGE | HMGB1/RAGE HMGB1/RAGE associated with the presence and severity of AAD-ALI, putative mechanism involving the activation of pro-inflammatory pathways. | **Clin:** Patient serum | ↑ | Elevated HMGB1 and RAGE in AAD-ALI vs AAD-nonALI (all *p* <0.05). Strong negative correlations with PaO₂/FiO₂: HMGB1 (r = -0.978) and RAGE (r = -0.944) (both *p* < 0.001). HMGB1 positively correlates with RAGE (*r* = 0.978, *p* < 0.001). | **Diagnostic.**Associated with the presence and severity of AAD-ALI. | **Clinical:** Level 3b (OCEBM) | (38) |
| TF | Independent predictor of AAD-ALI; putative mechanism as core initiator of coagulation cascade. | **Clin:** Patient serum and BALF | ↑ | Elevated TF and TFPI in serum and BALF (AAD-ALI vs AAD- nonALI, *P* < 0.001). Multivariable linear regression: serum β = -7.084, BALF β = -7.818, *p* < 0.001. | **Prognostic.** Independently predicted AAD-ALI. | **Clinical:** Level 2a (OCEBM) | (40) |

**VE-cadherin,** vascular endothelial cadherin**;** PMVECs, pulmonary microvascular endothelial cells; pY685-VE-cadherin, phospho-Y685-vascular endothelial cadherin;HMGB1, high mobility group box 1; RAGE, receptor for advanced glycation end-products; AAD-ALI, acute aortic dissection with acute lung injury; AAD-nonALI, acute aortic dissection patients without acute lung injury; OCEBM, Oxford Centre for Evidence-Based Medicine; TF, tissue factor; BALF, bronchoalveolar lavage fluid; TFPI, tissue factor pathway inhibitor.

**Supplemental TABLE 2 Candidate biomarkers for acute aortic dissection-induced acute lung injury/acute respiratory distress syndrome (Part 5 of 5)**

| **Biomarker** | **Pathway / Mechanism** | **Specimen** | **Direction** | **Key Reported Findings** | **Diagnostic / Prognostic performance** | **Study Design/ Level of evidence** | **Ref.** |
| --- | --- | --- | --- | --- | --- | --- | --- |
| PAI-1 | Independent predictor of AAD- ALI; putative role of lung-derived PAI-1 in promoting pulmonary fibrin deposition. | **Clin:** Patient serum and BALF | ↑ | Eelevated PAI-1 in serum and BALF of AAD-ALI vs. AAD-nonALI (*P* < 0.001), with BALF levels > serum. Strong negative correlation with PaO₂/FiO₂ (r = -0.978, *p* < 0.001). Multivariable linear regression: β = -11.229, *p* < 0.001. | **Prognostic.** Strongest independent predictor of AAD-ALI (model adjusted R² = 0.786). | **Clinical:** Level 2a (OCEBM) | (40) |
| MPV/platelet count ratio | Higher MPV/platelet count associated with higher AAD-Hypox risk; putative mechanisms include amplification of thrombo-inflammatory responses. | **Clin:** Patient serum | ↑ | Significantly higher hypoxemia with high MPV/ platelet count ratio in AAD (67.7% vs. 46.7%; *P*=0.048). | **Prognostic.** Predicts increased AAD-Hypox risk (cut-off >7.49). | **Clinical:** Level 2a (OCEBM) | (59) |
| TXB2 | Elevated TXB2 associated with higher AAD-ALI risk; putative mechanisms include platelet aggregation/ vasoconstriction. | **Clin:** Patient serum | ↑ | Elevated TXB2 in AAD-ALI vs. AAD-nonALI (*p* < 0.05). | **Diagnostic.**Suggests association between elevated TXB2 and higher AAD-ALI risk. | **Clinical:** Level 4 (OCEBM) | (60) |
| PGI2)/TXB2 ratio | Independent predictors for AAD-ALI; putative mechanisms include platelet aggregation/ vasoconstriction. | **Clin:** Patient serum | ↓ | Multivariate analysis identified low PGI₂/TXB₂ ratio (OR 0.25) as independent factor significantly associated with AAD-ALI. | **Diagnostic.** Independently associated with reduced AAD-ALI risk. | **Clinical:** Level 4 (OCEBM) | (60) |

PAI-1, plasminogen activator inhibitor-1; AAD-ALI, acute aortic dissection with acute lung injury; AAD-nonALI, acute aortic dissection patients without acute lung injury; BALF, bronchoalveolar lavage fluid; OCEBM, Oxford Centre for Evidence-Based Medicine; MPV, mean platelet volume; AAD-Hypox, AAD patients with hypoxemia; TXB2, thromboxane B2; PGI2, prostacyclin; TXA2, thromboxane A2.
