# Supplementary material for: Acute aortic dissection-induced acute respiratory distress syndrome: pathogenesis and clinical implications
Source: Front Cardiovasc Med. 2025 Nov 21;12:1654456. doi: 10.3389/fcvm.2025.1654456 (PMC12679279; doi:10.3389/fcvm.2025.1654456)
Supplement: Supplementary file 3 [file Datasheet1.pdf]

Literature search strategy

To comprehensively explore the pathogenesis and clinical implications of acute respiratory distress syndrome (ARDS) secondary to acute aortic dissection (AAD), a structured yet flexible search strategy was designed, adhering to narrative review principles. Studies were selected through a PRISMA-guided process. (Supplemental Figure 1)

1. Data sources

Electronic Databases: PubMed, Web of Science, Embase, and Cochrane Library

Gray Literature: Preprints (medRxiv, bioRxiv), conference abstracts (American Association for Thoracic Surgery, European Society of Cardiology).

Supplementary Sources: Reference lists of key reviews and guidelines.

2. Search terms

Search terms were constructed based on key concepts related to the research topic, including "*aortic dissection*", "*acute lung injury*", "*respiratory distress syndrome*" and "*hypoxia*." To enhance search sensitivity, synonyms, related terms, and Medical Subject Headings (MeSH) were used.

Search strategy example (PubMed):

In PubMed, the following search strategy was employed: *((((((((((((("Aortic Dissection"[Mesh]) OR (Aortic Dissections)) OR (Dissection, Aortic)) OR (Aneurysm, Dissecting)) OR (Dissecting Aneurysms)) OR (Dissecting Aneurysm)) OR (Dissecting Aneurysm Aorta)) OR (Aneurysm Aorta, Dissecting)) OR (Aorta, Dissecting Aneurysm)) OR (Dissecting Aneurysm Aortas)) OR (Aortic Dissecting Aneurysm)) OR (Aneurysm, Aortic Dissecting)) OR (Aortic Dissecting Aneurysms)) OR (Dissecting Aneurysm, Aortic)) AND*

((((("Acute Lung Injury"[Mesh]) OR (Acute Lung Injuries)) OR (Lung Injuries, Acute)) OR (Lung Injury, Acute)) OR (((((((((((((("Respiratory Distress Syndrome"[Mesh]) OR (Distress Syndrome, Respiratory)) OR (Distress Syndromes, Respiratory)) OR (Respiratory Distress Syndromes)) OR (Syndrome, Respiratory Distress)) OR (Shock Lung)) OR (Lung, Shock)) OR (Respiratory Distress Syndrome, Adult)) OR (Adult Respiratory Distress Syndrome)) OR (Respiratory Distress Syndrome, Acute)) OR (Acute Respiratory Distress Syndrome)) OR (ARDS, Human)) OR (Human ARDS)) OR (Respiratory Distress Syndrome, Pediatric)) OR (Pediatric Respiratory Distress Syndrome))) OR (((((((("Hypoxia"[Mesh]) OR (Deficiency, Oxygen)) OR (Deficiencies, Oxygen)) OR (Oxygen Deficiencies)) OR (Oxygen Deficiency)) OR (Anoxemia)) OR (Hypoxemia)) OR (Anoxia))) . The search was filtered to include English-language publications published from database inception to February 1, 2025.

3. Inclusion and exclusion criteria

Studies were included if they: 1) population: ARDS occurring in AAD patients before surgery; 2) study types: included clinical trials, observational studies, mechanistic studies (human/animal models, *in vitro* studies), and relevant reviews; 3) outcomes focusing: pathophysiological pathways, biomarkers, or therapeutic interventions.

Exclusion criteria comprised: 1) studies focusing solely on postoperative ARDS without preoperative relevance; 2) studies with insufficient data for analysis; 3) clinical studies with fewer than 50 cases; 4) letters, comments, case reports, or conference abstracts.

4. Study selection

1,208 were identified from the selected databases. After removing duplicates (n = 432), two authors independently screened the titles and abstracts of 776 identified records. Full texts

of potentially eligible studies were assessed for mechanistic or clinical relevance. Discrepancies resolved through consensus or third-party adjudication. Full texts of 69 potentially relevant articles were assessed, with 34 studies meeting the eligibility criteria (excluded: studies focusing solely on postoperative ARDS without preoperative relevance [n = 31], letter, comments, case reports, conference abstracts [n = 2], clinical studies with fewer than 50 cases [n = 1]. Manual backward snowballing (screening references of included studies) added 2 articles.

5. Data extraction

Key variables were cataloged:

Clinical Data: ARDS incidence, risk factors (e.g., CRP levels, BMI), mortality rates, mechanical ventilation time, ICU stay, hospital stay.

Pathogenesis: Such as inflammatory mediators (e.g., IL-6, TNF-α), Ang II, HMGB1, and tissue factor, as well as histopathological evidence.

Interventions: Respiration assistance, medical treatment, potential intervention measures (e.g., MCP-1 inhibitor).

6. Quality appraisal

Clinical studies were evaluated using the ROBINS-I tool for bias risk. Mechanistic studies prioritized those with experimental validation.

7. Limitations

Potential language bias (non-English studies excluded). Heterogeneity in ARDS diagnostic criteria and across studies. This strategy balanced comprehensiveness and narrative synthesis goals, enabling critical integration of multidisciplinary evidence.
