# Supplementary material for: Acute aortic dissection-induced acute respiratory distress syndrome: pathogenesis and clinical implications
Source: Front Cardiovasc Med. 2025 Nov 21;12:1654456. doi: 10.3389/fcvm.2025.1654456 (PMC12679279; doi:10.3389/fcvm.2025.1654456)
Supplement: Supplementary file 4 [file Image1.pdf]

Supplemental Figure 1. PRISMA flowchart of literature search and study selection.

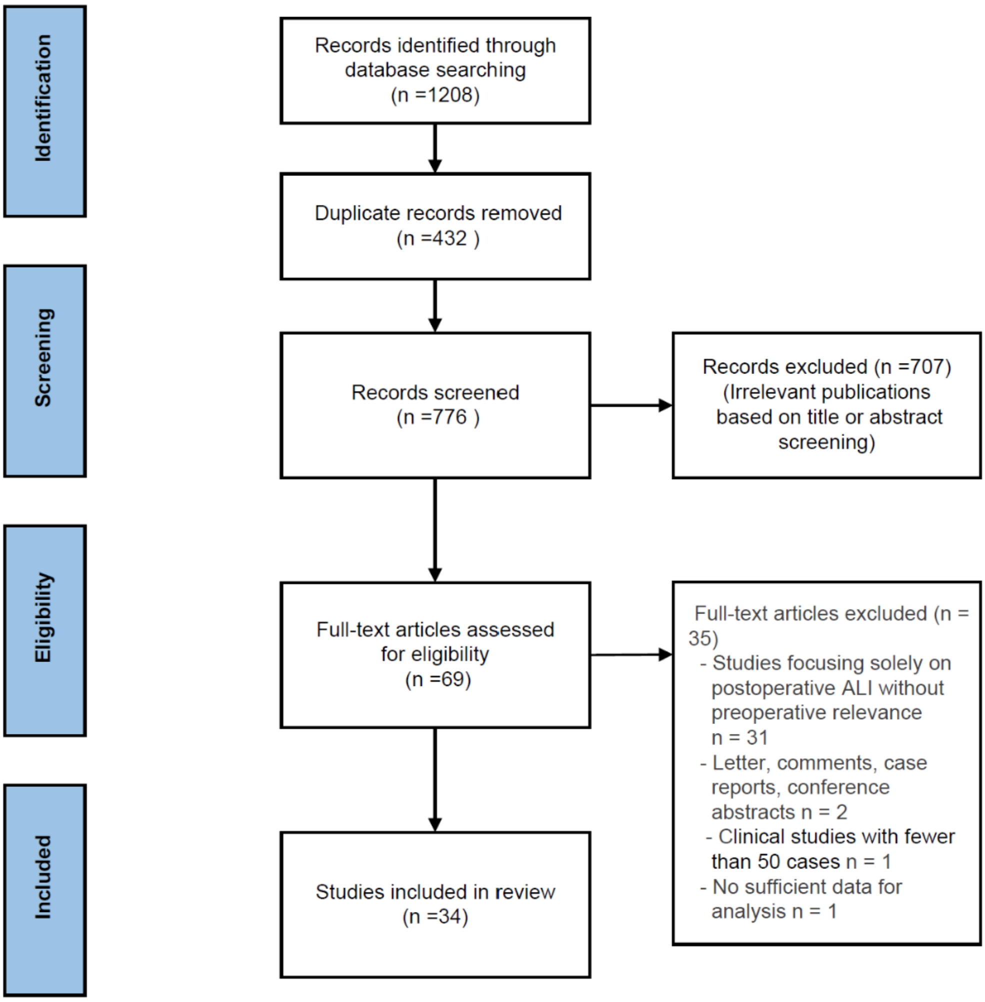

Supplemental Figure 1. Flowchart summarizing the literature search and selection process for studies on acute aortic dissection (AAD)-induced acute respiratory distress syndrome (ARDS). A total of 1,208 records were identified through PubMed, Web of Science, Embase, and Cochrane Library. After removal of 432 duplicates, 776 titles/abstracts were independently screened by two authors. Full texts of 69 potentially eligible articles were assessed for mechanistic insights and clinical relevance. Discrepancies were resolved through consensus or third-party adjudication, resulting in 34 studies included in the final analysis (excluded: studies focusing solely on postoperative ARDS without preoperative relevance [n = 31], letter, comments, case reports, conference abstracts [n = 2], clinical studies with fewer than 50 cases [n = 1], no sufficient data for analysis [n = 1]).
